# Face-Down Posture versus Non-Face-Down Posture following Large Idiopathic Macular Hole Surgery: A Systemic Review and Meta-Analysis

**DOI:** 10.3390/jcm10214895

**Published:** 2021-10-24

**Authors:** Hou-Ren Tsai, Tai-Li Chen, Chun-Yu Chang, Huei-Kai Huang, Yuan-Chieh Lee

**Affiliations:** 1 Department of Medical Education, Medical Administration Office, Hualien Tzu Chi Hospital, Buddhist Tzu Chi Medical Foundation, Hualien 970, Taiwan; melotsai0830@gmail.com (H.-R.T.); terrychen.a@gmail.com (T.-L.C.); 2 School of Medicine, Tzu Chi University, Hualien 970, Taiwan; paulchang1231@gmail.com (C.-Y.C.); drhkhuang@gmail.com (H.-K.H.); 3 Department of Dermatology, Tzu Chi Skin Institute, Hualien Tzu Chi Hospital, Buddhist Tzu Chi Medical Foundation, Hualien 970, Taiwan; 4 Department of Anesthesiology, Taipei Tzu Chi Hospital, Buddhist Tzu Chi Medical Foundation, New Taipei 231, Taiwan; 5 Institute of Epidemiology and Preventive Medicine, College of Public Health, National Taiwan University, Taipei 100, Taiwan; 6 Department of Medical Research, Hualien Tzu Chi Hospital, Buddhist Tzu Chi Medical Foundation, Hualien 970, Taiwan; 7 Department of Family Medicine, Hualien Tzu Chi Hospital, Buddhist Tzu Chi Medical Foundation, Hualien 970, Taiwan; 8 Department of Ophthalmology, Buddhist Tzu Chi General Hospital, Hualien 970, Taiwan; 9 Department of Ophthalmology and Visual Science, Tzu Chi University, Hualien 970, Taiwan

**Keywords:** face-down posture, idiopathic macular holes, systemic review

## Abstract

Evidence regarding the effect of a face-down posture (FDP) for large idiopathic macular hole (IMH) is inconsistent. We conducted a systematic review and meta-analysis to determine whether a postoperative FDP is required for the treatment of large IMH. Eligible randomized controlled trials published before September 2021 were retrieved from the Medline, Embase, and Cochrane Library databases. The efficacy outcome was the IMH closure rate and the visual acuity improvement rate. A meta-analysis was performed using a random effects model. The “Grading of Recommendations Assessment, Development, and Evaluation” approach was implemented, and the numbers needed-to-treat (NNTs) were calculated. Seven studies comprising 640 patients were included. We performed a predefined subgroup analysis of IMH size using a cut-off point of 400 µm. Compared with non-FDP, a significant effect of FDP was found in the IMH > 400 µm group (OR = 3.34; 95% CI = 1.57–7.14; trial sequential analysis-adjusted CI = 1.20–11.58; NNTs = 7.9). After stratifying by the posturing periods, the beneficial effect of FDP lasting at least five days, but not three days was observed for large IMH. Maintaining a FDP for at least five days postoperatively is an effective strategy (certainty of evidence: “moderate”) for treating large IMH.

## 1. Introduction

Idiopathic macular hole (IMH) is a significant cause of sight impairment in older people, with an incidence of 7.9 per 100,000 individuals per year [1,2]. It primarily forms due to anteroposterior vitreomacular traction and the proliferation of glial cells [3,4]. Surgical management for IMH includes vitrectomy, internal limiting membrane (ILM) peeling or peeling/inverting, and gas tamponade. Following IMH surgery, maintaining a face-down posture (FDP) for a minimum of one week has been recommended to ensure continuous gas–fovea contact [5]. According to a survey by the American Society of Retina Specialists in 2018 [6], 86.9% of retinal surgeons incorporate the FDP in clinical practice. However, maintaining a FDP is arduous, particularly for older and obese people, and may lead to some complications such as pulmonary embolism, ulnar nerve neuropathy, and pressure sore [7,8,9].

More recent studies have focused on the need for the FDP in patients receiving IMH surgery. Previous studies have proven that maintaining a FDP is unnecessary for cases with small IMH (<400 µm) [10,11,12]. However, evidence regarding the effect of the FDP on large IMH (>400 µm) [11,12,13] has been inconsistent, and most randomized controlled trials (RCTs) have involved a small number of cases. Although previous meta-analyses [14,15,16] have pooled the published results to yield more reliable evidence, the number of enrolled studies in each meta-analysis was relatively small, and thus lacked statistical power to draw solid conclusions [17]. A recent meta-analysis [18] enrolled 11 studies to increase statistical power. However, it included case–control, non-randomized, and historically controlled cohort studies, which may have introduced information and selection bias. Thus, a comprehensive literature review with meta-analysis is warranted for better understanding and compilation of the currently available evidence. 

Consequently, we here conducted a systematic review and meta-analysis to evaluate the effects of maintaining a FDP following large IMH surgery. We also graded the certainty of evidence (CoE) based on the “Grading of Recommendations Assessment, Development and Evaluation” (GRADE) approach and calculated the numbers needed-to-treat (NNTs) to facilitate clinical decision making.

## 2. Materials and Methods

### 2.1. Study Design

This meta-analysis aimed to evaluate the effect of postoperative FDP on large macular-hole surgery (MHS). The study was conducted according to the recommendations of the Preferred Reporting Items for Systematic Review and Meta-analysis (PRISMA) statement (Table S1). The methodology was pre-specified, and the study was registered on the PROSPERO website (Registration No. CRD42021265714).

### 2.2. Search Strategy

Studies investigating the effect of postoperative FDP in patients with IMH before September 2021 were identified from the Medline, Embase, and Cochrane Library databases. No language restrictions were imposed. The keywords “macular hole,” “face down,” and “prone position,” as well as their synonyms and derivatives, were used. The details of the search strategies are presented in Table S2. We further scrutinized the reference lists of the included articles to identify other relevant studies. 

### 2.3. Inclusion and Exclusion Criteria

Two authors (HRT and TLC) independently identified relevant studies by screening the titles and abstracts of the papers identified in the literature search. The full texts of potentially relevant articles were obtained and examined for eligibility. Any discrepancy was resolved through discussion with a third author (YCL). Studies were included in the systematic review if they satisfied the following criteria: they were RCTs, they compared FDP versus non-FDP (nFDP) after IMH surgery, and they reported at least one efficacy outcome relevant to our review, including the IMH closure rate or visual acuity (VA) improvement rate. We excluded review articles, case reports, case series, and animal or laboratory studies.

### 2.4. Data Extraction

Two authors (HRT and TLC) independently extracted the following items: first author, year of publication, study design, number of cases, follow-up period, definition of VA improvement, gas used, ILM peeling, and posturing protocol. The primary outcome was the IMH closure rate, and the secondary outcome was the VA improvement rate. In cases where specific aspects required clarification, efforts were made to contact the corresponding authors of the relevant papers for further information. 

### 2.5. Quality Assessment

The methodological quality of included RCTs was evaluated using the Cochrane Collaboration’s Risk-of-Bias Assessment tool (RoB v.2.0) [19]. Decisions individually recorded by the reviewers (HRT and TLC) were compared, and disagreements were resolved by a third reviewer (YCL).

### 2.6. Data Synthesis and Statistical Analyses

The meta-analysis was performed using Stata v17 (StataCorp, College Station, TX, USA). The effect size of each study was presented as an odds ratio (OR) with 95% confidence intervals (CIs) for dichotomous outcome measures (IMH closure rate and VA improvement rate). The pooled estimates and their CIs were calculated using the DerSimonian and Laird random-effects model, considering the heterogeneity of the study protocols [20]. Statistical heterogeneity among studies was tested using the I^2^ and Cochran Q statistics [21]. Statistical heterogeneity was considered substantial when the I^2^ statistic was ≥ 50% and if *p* < 0.01 for Q. The pooled effect sizes were deemed significant when the 95% CI of the OR did not span 1. All statistical tests were two-sided, and *p* values < 0.05, were considered statistically significant. 

For the IMH closure rate, we conducted a predefined subgroup analysis of IMH size using 400 µm as a cut-off point, according to the International Vitreomacular Traction Study Group Classification [22]. The effect of FDP was assessed for large IMH (>400 µm). Potential effect modifiers, such as the length of the posturing period and of the follow-up period, were also evaluated. 

A leave-one-out sensitivity analysis was performed to assess the influence of each study on the overall effect by sequentially removing studies. The Doi plot with the Luis Furuya-Kanamori (LFK) index was used to evaluate publication bias for large IMH [23]. If values of the LFK index fell outside the interval between −1 and +1, the Doi plot was considered asymmetrical, which may indicate publication bias. 

### 2.7. Trial Sequential Analysis

Meta-analyses may be subject to an increased risk of type-1 and type-2 errors due to sparse data and repetitive testing of accumulating data [24,25]. Therefore, if the data were too sparse to confirm the conclusions, trial sequential analysis (TSA) was used to challenge the meta-analysis and to avoid early overestimates by combining the estimated required information size with an adjusted threshold [26]. According to the O’Brien–Fleming alpha-spending function, we constructed TSA boundaries to determine whether the *p* value was sufficiently statistically significant to show the anticipated effect, or whether the analysis should be terminated early [27]. We adjusted the relevant parameters according to the RCT of Pasu et al. [28]. A 5% (α = 0.05; two-sided) total risk of type-1 error and 85% statistical power were set, and a relative risk difference of 15% was set for defining improved large IMH closure rate. The event rate in the control group was 80%. We provided TSA-adjusted 95% CIs. Random-effect TSA was performed using TSA software (version 0.9.5.10 Beta; Copenhagen Trial Unit, Copenhagen, Denmark).

### 2.8. Grading of Certainty of Evidence

The GRADE approach was adopted to provide the CoE at the outcome level [29] and was evaluated by all authors. Evidence was classified as high, moderate, low, or very low quality, based on the RoB, consistency, directness, precision, and publication bias. In the event of disagreement, discussions were conducted until a consensus was reached for each outcome. NNTs were calculated to evaluate the evidence-based effect of postoperative FDP in patients with IMH. 

## 3. Results

### 3.1. Search Results

**Figure 1:**
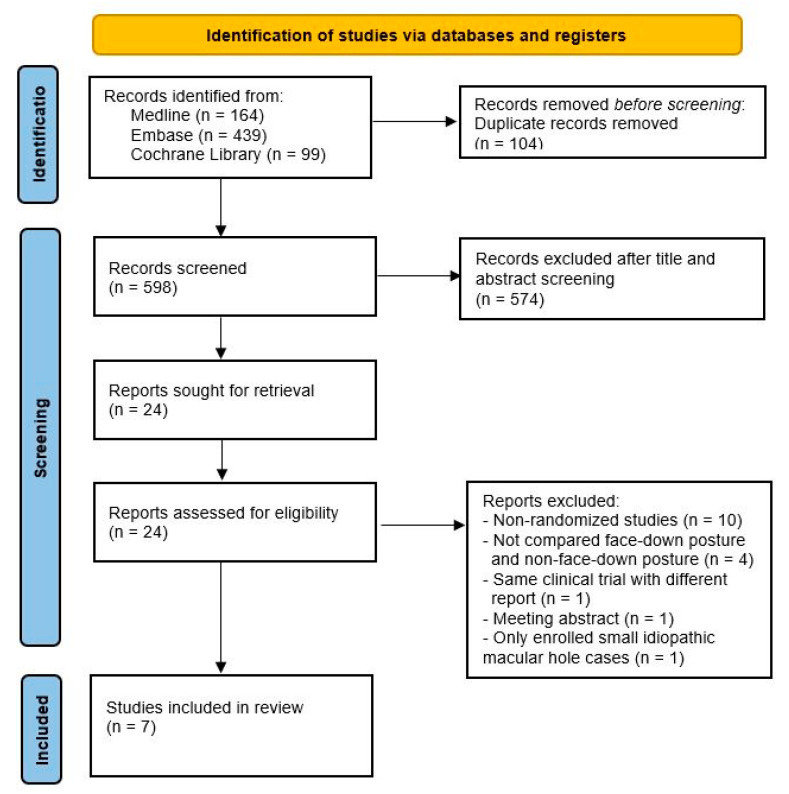
PRISMA flow diagram.

Figure 1 presents a PRISMA flow diagram outlining the screening and selection of the included studies. We identified 164, 439, and 99 articles from the Medline, Embase, and Cochrane libraries, respectively. After eliminating 104 duplicate articles, 598 articles remained. After screening the titles and abstracts, 574 articles were excluded. The remaining 24 studies underwent full-text screening. Table S3 lists the details of the excluded studies. Seven RCTs from 2008 to 2020 were included in the final meta-analysis.

### 3.2. Study Characteristics

The basic characteristics of the included studies are summarized in Table 1. A total of 371 participants with large IMH (FDP, 183; nFDP, 188) were included in our study. Six studies [11,12,13,28,30,31,32] were conducted in Europe, and one was conducted [9] in Asia. The FDP protocols included 3 [13,31,32], 5 [11,28], and 10 [12,30] days of FDP. The mean age of the patients ranged from 62.35 to 72.4 years. Regarding the follow-up period, three RCTs [13,28,32] involved a 3-month follow-up, whereas three [11,30,31] used a 6-month follow-up, and one [12] followed patients up for 6–8 weeks. Notably, Veith et al. [31]. divided patients into four arms according to the intraocular tamponade (air or SF6) and the use of FDP. We only extracted data from the air group because the data in the SF6 group were incomplete.

### 3.3. Risk-of-Bias Assessment

Most domain-level judgments in the enrolled RCTs indicated a low RoB. In the domain of randomization, two enrolled trials [11,31] were judged as presenting “some concerns” because the process of allocation concealment was not clearly described. With regard to the domain of deviation from the intended intervention, two trials [12,30] were judged as “some concerns,” because the way they informed patients on how to conduct the FDP was not mentioned. As for the domain of missing outcome data, measurement of outcome, and selection of the reported result, the seven enrolled trials had a low RoB. The detailed RoB for the enrolled RCTs is shown in Table S4.

### 3.4. Pooled Effects of the Large IMH Closure Rate

**Figure 2:**
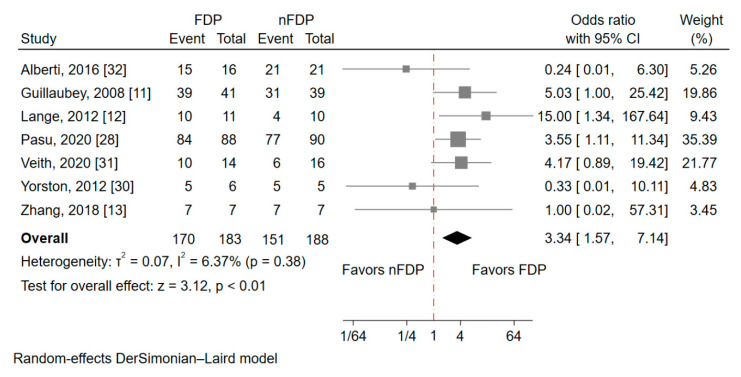
Forest plot of large idiopathic macular hole closure rate.

Seven RCTs, including 183 and 188 patients in the FDP and nFDP groups, respectively, reported the outcome of the closure rate for large IMH. Meta-analysis results demonstrated that IMH patients who adhered to the FDP had higher odds of achieving IMH closure than those in the nFDP group (OR = 3.34; 95% CI = 1.57–7.14; *p* < 0.01) (Figure 2). The overall heterogeneity I^2^ was 6.37% (*p* = 0.38). Leave-one-out sensitivity analysis yielded similar results, demonstrating the robustness of the findings (Figure S1).

### 3.5. Subgroup Analysis

We further evaluated the effects of the posturing period and the follow-up period (Table 2). The results demonstrated that FDP significantly increased the odds of IMH closure in the group with a posturing period ≥ 5 days and a follow-up period of ≥6 months, whereas no significant effect was observed in the group with a posturing period ≤ 3 days and a follow-up period ≤ 3 months.

### 3.6. Trial Sequential Analysis

**Figure 3:**
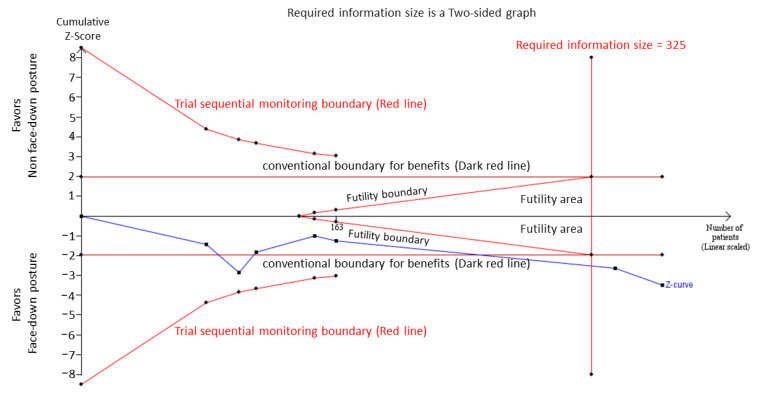
Trial sequential analysis of large idiopathic macular hole closure rate.

Figure 3 shows the TSA of the large IMH closure rate. The cumulative Z-curve surpassed the traditional boundary for statistical significance after the inclusion of Peru et al. and crossed the trial sequential monitoring boundary after Veith et al. for deriving the benefit of FDP, suggesting conclusive results (TSA-adjusted CI: 1.20–11.58). The RIS value of 325 was also reached. 

### 3.7. Pooled Effects of the Visual Acuity Improved Rate

Four RCTs, including 153 patients in the FDP group and 153 in the nFDP group, analyzed the effect of a FDP on the VA improvement rate (Figure S2). The pooled results showed that IMH patients who adhered to the FDP did not have higher odds of achieving a VA improvement rate than those in the nFDP group (OR = 1.20; 95% CI = 0.48–3.03; *p* = 0.69), and significant heterogeneity was found (I^2^ = 54.46 %; *p* = 0.09). After omitting the papers individually in a sensitivity analysis, the ORs were found to be similar to the above findings (Figure S3). 

### 3.8. Publication Bias

The Doi plot demonstrated major asymmetry with an LFK index of −2.57 (Figure S4).

### 3.9. GRADE Approach for CoE

The GRADE assessments are summarized in Table S5, and the quality of evidence for the efficacy outcome was “moderate.” We downgraded the overall CoE in the domain of publication bias because minor asymmetry was observed in the Doi plot. The NNT for patients to achieve IMH closure was 7.9. 

## 4. Discussion

In this large-scale meta-analysis involving 640 participants from seven RCTs, we found a significant effect of FDP on the IMH > 400 µm group (OR = 3.34; 95% CI = 1.57–7.14; TSA-adjusted CI = 1.20–11.58; NNTs = 7.9) compared with the nFDP regarding IMH closure rate. The results were conclusive after post-hoc TSA. We found significant effects in the group with posturing periods ≥5 days and follow-up periods ≥6 months. However, IMH patients who adhered to the FDP did not have higher odds of achieving aVA improvement (OR = 1.20; 95% CI = 0.48–3.03).

One previous meta-analysis [15], which synthesized data from 227 participants in four RCTs published before 2015, demonstrated lower IMH closure rates in the nFDP group (OR = 0.33; 95% CI = 0.13–0.81). However, this study failed to indicate the effect of the FDP for large IMH due to the limitations of the data. In 2019, another meta-analysis [14], involving 358 participants from five RCTs, also demonstrated that the overall IMH closure rate in the FDP group was significantly higher than that in the nFDP group (OR = 2.27; 95% CI = 1.02–5.05). Further subgroup analysis indicated that the FDP may have more benefits for IMH larger than 400 µm, while most studies included in the analysis had small sample sizes, and multicenter RCTs are lacking. Considering the above points, our present meta-analysis used updated evidence, including seven RCTs, and found that FDP was beneficial for IMH > 400 µm. We used statistical methods to obtain precise results. TSA and Doi plots were used as adjunctive tools for an advanced and rigorous rating of the CoE in GRADE, because a previous study [33] reported that adoption of TSA leads to more frequent downgrading of the CoE, and that the LFK index of the Doi plot demonstrates higher sensitivity to publication-bias evaluation than Egger’s regression test [23]. Notably, Z curves for the large IMH group crossed the O’Brien–Fleming boundaries and RIS lines after inclusion of the trial by Pasu et al. [28]. We considered that these results may support our hypothesis that most previous studies, including RCTs and meta-analysis, involve only a small number of cases. We also applied a GRADE assessment and provided the NNT results to enable evidence-based decision-making in clinical practice. Hence, our updated meta-analysis is robust and provides more evidence than previously published meta-analyses.

How long a FDP should be implemented after macular hole surgery is debated. In contrast to a previous meta-analysis [14] that suggested that patients with IMH > 400 µm should maintain a FDP for 3 days after IMH surgery, our subgroup analysis found a significant effect of FDP only when maintained for at least 5 days, but not for a FDP maintained for only 3 days. Our results support the findings of previous studies, [34,35] which recommended maintaining the prone position for at least 5 days to maximize the contact of the bubble with the macular landscape. Additionally, in a retrospective study of 32 eyes, Shah et al. [36]. reported that large holes (>400 µm) may require FDP for 6 days to achieve anatomical success, based on spectral-domain optical coherence tomography. The distance between the broken ends of an IMH > 400 µm is large, and an IMH of this size is a significant risk factor for failure of IMH closure [37]. The absorption of gas with time may decrease the surface tension acting on the gas–macula contact area [38,39]. Thus, we asserted that our result supporting an extended posturing period of at least 5 days was reasonable.

The NNTs refer to the number of patients that need to undergo a particular therapy to achieve one additional positive outcome as compared with the placebo [40]. An NNT < 10 indicates the “clinically desirable” benefit of a particular therapeutic intervention [41]. In this study, the NNTs for the IMH > 400 μm was 7.9, indicating desirable effects as compared with nFDP. We believe that our findings could help clinical ophthalmologists treat patients with different IMH sizes effectively.

We found no significant effect of a FDP on the VA improvement rate. This discrepancy between the anatomical and functional results can be explained by several assumptions. Many factors are associated with visual success after IMH surgery, including preoperative VA, IMH size, duration of IMH before surgery, shape of macular closure, and the macular-hole index, defined as the ratio of the hole height to the base diameter [42,43,44]. Owing to the insufficient data in our included studies, we could not eliminate the confounding effects of associated factors and predict the potential role of the FDP in the process of visual recovery. Ittarat et al. [42] pointed out that VA outcomes are not always satisfactory, even when there is anatomical success. The most important predictors of postoperative VA may be foveal photoreceptor defects and the thickness of the outer retinal layer [45]. Oh et al. [46] found that a post-operative ellipsoid zone (EZ) defect correlates with poor postoperative VA (*p* = 0.010). Using spectral-domain optical-coherence tomography, Chang et al. [47] demonstrated, through a retrospective case series, that postoperative VA correlated with resolved glial cells and a restored external limiting membrane (ELM) and EZ line. They also showed that the gradual recovery of the outer retinal layer may also continue for several years after IMH surgery [48,49]. Thus, although the anatomical closure of IMH is observed, the outer retinal layers may not be completely restored, thereby leading to unsatisfactory visual outcomes.

Our study had several limitations. First, although most authors in the enrolled studies used a cut-off point of 400 µm, we could not determine whether the beneficial effect of the FDP was more prominent in patients with a larger IMH. Second, we were unable to depict the actual mechanism of the FDP following IMH surgery, despite its potential effect on increased gas–foveal contact. Third, we did not conduct a subgroup analysis for the VA improvement rate due to insufficient data. Furthermore, the follow-up periods in most of our enrolled studies may have been insufficient to observe the effect of FDP on VA. Finally, one of our included studies incorporated the inverted flap technique into their clinical practice [13]. Although our leave-one-out sensitivity analysis remained stable after excluding this study, further studies may be necessary to evaluate the effect of FDP following IMH surgery with inverted flap technique.

In conclusion, this systematic review and meta-analysis updated current evidence and used robust methods to prove the beneficial effect of implementing a FDP following large IMH surgery. A FDP duration of at least 5 days was recommended for IMH > 400 µm. Future studies are necessary to investigate the effects of FDP on VA following large IMH surgery.

## Figures and Tables

**Table 1:** Characteristics of studies included in the meta-analysis.

Study (First Author, Year)	Country	Study Design	Posturing Protocol	Gas Use	ILM Peeling	Number of Cases	Mean Age (Years)		MH Size		Definition of Visual Acuity Improvement	Follow-Up Periods
							FDP	nFDP	<400 µm (F/N)	>400 µm (F/N)		
Alberti, 2016 [32]	Denmark	RCT	3 days; 16 h/day	C_3_F_8_	Yes	68	69.8	69.3	18/13	16/21	≥15 letters (ETDRS) gain	3 months
Guillaubey, 2008 [11]	France	RCT	5 days; 8 h/day	C_3_F_8,_ C_2_F_8,_ SF_6_	Yes	150	69.0	68.0	37/33	41/39	NA	6 months
Lange, 2012 [12]	UK	RCT	10 days; 50 min/h	C_3_F_8_	Yes	30	66.8	71.0	4/5	11/10	≥0.2 logMAR units gain	6–8 weeks
Pasu, 2020 [28]	UK	RCT	5 days; 8 h/day	C_3_F_8_	Yes	178	69	69	NA	90/88	≥0.3 logMAR units gain	3 months
Veith, 2020 [31]	Spain	RCT	3 days; 24 h/day	Air	Yes	51	69.2	71.3	12/9	14/16	NA	6 months
Yorston, 2012 [30]	UK	RCT	10 days; 50 min/h	C_3_F_8_	Yes	30	71.1	68.0	10/9	6/5	≥6/18 (Snellen)	6 months
Zhang, 2018 [13]	China	RCT	3 days; 16 h/day	C_3_F_8_	Yes	80	62.35	62.85	33/33	7/7	NA	3 months

Abbreviations: ETDRS Early Treatment Diabetic Retinopathy Study, FDP face-down posture, F FDP, ILM internal limiting membrane, MH macular hole, nFDP non-facedown posture, N nFDP, NA not applicable, RCT randomized controlled trial.

**Table 2:** Subgroup analyses of large IMH closure rate.

	Large IMH Closure Rate			
Subgroups	No. of Trials	Pooled OR (95% CI)	*p*-Value	I^2^ (%)
Posturing periods				
≤3 days	3	1.76 (0.32 to 9.70)	0.52	22.21
≥5 days	4	4.05 (1.60 to 10.22)	<0.01 *	9.30
Follow-up periods				
≤3 months	4	2.91 (0.71 to 11.98)	0.14	30.73
≥6 months	3	3.51 (1.18 to 10.39)	0.02 *	3.22

* *p* < 0.05; IMH, idiopathic macular hole.

## Data Availability

Data supporting the findings of this study are available within the included articles or published studies.

## Supplementary Materials

The following are available online at https://www.mdpi.com/article/10.3390/jcm10214895/s1, Table S1. PRISMA checklist. Figure S1. Leave-one-out sensitivity analysis of large IMH closure rate. Table S2. Search strategy modified in PubMed (a), Embase (b), Cochrane CENTRAL (c). Figure S2. Forrest plot of visual acuity improved rate. Table S3. References of excluded studies after full-text screening. Figure S3. Leave-one-out sensitivity analysis of visual acuity improved rate. Table S4. Summary of ROB 2.0 assessment in RCTs. Figure S4. DOI plot of large IMH closure rate. Table S5. GRADE assessment.
