# Supplementary material for: Face-Down Posture versus Non-Face-Down Posture following Large Idiopathic Macular Hole Surgery: A Systemic Review and Meta-Analysis
Source: J Clin Med. 2021 Oct 24;10(21):4895. doi: 10.3390/jcm10214895 (PMC8584827; doi:10.3390/jcm10214895)
Supplement: Supplementary file 1 [file jcm-10-04895-s001.zip › jcm-1415214-supplementary.pdf]

Supplementary Online Contents

Table S1. PRISMA checklist

Figure S1. Leave-one-out sensitivity analysis of large IMH closure rate

Table S2. Search strategy modified in PubMed (a), Embase (b), Cochrane CENTRAL (c)

Figure S2. Forrest plot of visual acuity improved rate

Table S3. References of excluded studies after full-text screening

Figure S3. Leave-one-out sensitivity analysis of visual acuity improved rate

Table S4. Summary of ROB 2.0 assessment in RCTs

Figure S4. DOI plot of large IMH closure rate

Table S5. GRADE assessment.

Table S1. PRISMA checklist

Section and Topic	Item #	Checklist item	Location where item is reported
**TITLE**			
Title	1	Identify the report as a systematic review.	P.1
**ABSTRACT**			
Abstract	2	See the PRISMA 2020 for Abstracts checklist.	P.1
**INTRODUCTION**			
Rationale	3	Describe the rationale for the review in the context of existing knowledge.	P.1-2
Objectives	4	Provide an explicit statement of the objective(s) or question(s) the review addresses.	P.2
**METHODS**			
Eligibility criteria	5	Specify the inclusion and exclusion criteria for the review and how studies were grouped for the syntheses.	P.2
Information sources	6	Specify all databases, registers, websites, organisations, reference lists and other sources searched or consulted to identify studies. Specify the date when each source was last searched or consulted.	P.2
Search strategy	7	Present the full search strategies for all databases, registers and websites, including any filters and limits used.	Table S2
Selection process	8	Specify the methods used to decide whether a study met the inclusion criteria of the review, including how many reviewers screened each record and each report retrieved, whether they worked independently, and if applicable, details of automation tools used in the process.	P.2
Data collection process	9	Specify the methods used to collect data from reports, including how many reviewers collected data from each report, whether they worked independently, any processes for obtaining or confirming data from study investigators, and if applicable, details of automation tools used in the process.	P.2-3
Data items	10a	List and define all outcomes for which data were sought. Specify whether all results that were compatible with each outcome domain in each	P.2

Section and Topic	Item #	Checklist item	Location where item is reported
		study were sought (e.g. for all measures, time points, analyses), and if not, the methods used to decide which results to collect.	
	10b	List and define all other variables for which data were sought (e.g. participant and intervention characteristics, funding sources). Describe any assumptions made about any missing or unclear information.	Nil
Study risk of bias assessment	11	Specify the methods used to assess risk of bias in the included studies, including details of the tool(s) used, how many reviewers assessed each study and whether they worked independently, and if applicable, details of automation tools used in the process.	P.3
Effect measures	12	Specify for each outcome the effect measure(s) (e.g. risk ratio, mean difference) used in the synthesis or presentation of results.	P.3
Synthesis methods	13a	Describe the processes used to decide which studies were eligible for each synthesis (e.g. tabulating the study intervention characteristics and comparing against the planned groups for each synthesis (item #5)).	P.2-3
	13b	Describe any methods required to prepare the data for presentation or synthesis, such as handling of missing summary statistics, or data conversions.	P.2
	13c	Describe any methods used to tabulate or visually display results of individual studies and syntheses.	Nil
	13d	Describe any methods used to synthesize results and provide a rationale for the choice(s). If meta-analysis was performed, describe the model(s), method(s) to identify the presence and extent of statistical heterogeneity, and software package(s) used.	P.3
	13e	Describe any methods used to explore possible causes of heterogeneity among study results (e.g. subgroup analysis, meta-regression).	P.3
	13f	Describe any sensitivity analyses conducted to assess robustness of the synthesized results.	P.3
Reporting bias assessment	14	Describe any methods used to assess risk of bias due to missing results in a synthesis (arising from reporting biases).	P.3
Certainty assessment	15	Describe any methods used to assess certainty (or confidence) in the body of evidence for an outcome.	P.3
**RESULTS**			
Study selection	16a	Describe the results of the search and selection process, from the number of records identified in the search to the number of studies included in the review, ideally using a flow diagram.	P.4

Section and Topic	Item #	Checklist item	Location where item is reported
	16b	Cite studies that might appear to meet the inclusion criteria, but which were excluded, and explain why they were excluded.	Table S3.
Study characteristics	17	Cite each included study and present its characteristics.	Table 1.
Risk of bias in studies	18	Present assessments of risk of bias for each included study.	Table S4.
Results of individual studies	19	For all outcomes, present, for each study: (a) summary statistics for each group (where appropriate) and (b) an effect estimate and its precision (e.g. confidence/credible interval), ideally using structured tables or plots.	Figure 2.
Results of syntheses	20a	For each synthesis, briefly summarise the characteristics and risk of bias among contributing studies.	P.4
	20b	Present results of all statistical syntheses conducted. If meta-analysis was done, present for each the summary estimate and its precision (e.g. confidence/credible interval) and measures of statistical heterogeneity. If comparing groups, describe the direction of the effect.	Figure 2. Figure S2. P.6
	20c	Present results of all investigations of possible causes of heterogeneity among study results.	P.6-7
	20d	Present results of all sensitivity analyses conducted to assess the robustness of the synthesized results.	Figure S1. Figure S3.
Reporting biases	21	Present assessments of risk of bias due to missing results (arising from reporting biases) for each synthesis assessed.	Figure S4. P.6
Certainty of evidence	22	Present assessments of certainty (or confidence) in the body of evidence for each outcome assessed.	Table S5.
**DISCUSSION**			
Discussion	23a	Provide a general interpretation of the results in the context of other evidence.	P.8
	23b	Discuss any limitations of the evidence included in the review.	P.9

Section and Topic	Item #	Checklist item	Location where item is reported
	23c	Discuss any limitations of the review processes used.	P.9
	23d	Discuss implications of the results for practice, policy, and future research.	P.9
**OTHER INFORMATION**			
Registration and protocol	24a	Provide registration information for the review, including register name and registration number, or state that the review was not registered.	P.2
	24b	Indicate where the review protocol can be accessed, or state that a protocol was not prepared.	P.3
	24c	Describe and explain any amendments to information provided at registration or in the protocol.	Nil
Support	25	Describe sources of financial or non-financial support for the review, and the role of the funders or sponsors in the review.	P.10
Competing interests	26	Declare any competing interests of review authors.	P.10
Availability of data, code and other materials	27	Report which of the following are publicly available and where they can be found: template data collection forms; data extracted from included studies; data used for all analyses; analytic code; any other materials used in the review.	Nil

Figure S1. Leave-one-out sensitivity analysis of large IMH closure rate

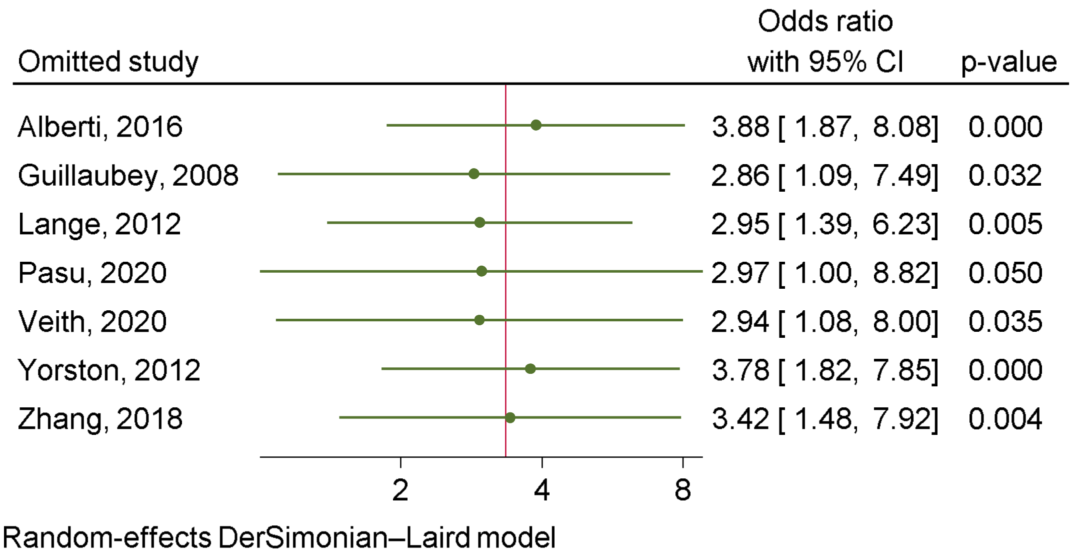

Table S2. Search strategies modified in Medline (a), Embase (b), and Cochrane CENTRAL (c)

a. Search strategy in Medline (via Ovid MEDLINE, 1946–present; search date: 2021/09/05)

#	Search syntax	Citations found
1	prone position/	
2	exp postoperative period/	
3	exp postoperative care/	
4	(postur$ or position$ or prone).tw.	
5	(face adj3 down).tw.	
6	or/1-5	
7	exp retinal perforations/	
8	(macula$ adj4 hole$).tw.	
9	or/7-8	
10	randomized controlled trial.pt.	
11	controlled clinical trial.pt.	
12	randomly.ab,ti.	
13	(randomized or randomised).ab,ti.	
14	placebo.ab,ti.	
15	trial.ab,ti.	
16	groups.ab,ti.	
17	dt.fs.	
18	or/10-17	

19	6 and 9 and 18	164

b. Search strategy in Embase (via Ovid, 1974–present; search date: 2021/09/05)

#	Search syntax	Citations found
1	exp randomized controlled trial/	
2	exp randomization/	
3	exp double blind procedure/	
4	exp single blind procedure/	
5	random$.tw.	
6	or/1-5	
7	(animal or animal experiment).sh.	
8	human.sh.	
9	7 and 8	
10	7 not 9	
11	6 not 10	
12	exp clinical trial/	
13	((singl$ or doubl$ or trebl$ or tripl$) adj3 (blind$ or mask$)).tw.	
14	random$.tw.	
15	placebo$.tw.	
16	exp placebo/	
17	(clin$ adj3 trial$).tw.	
18	or/12-17	
19	18 not 10	
20	18 not 11	
21	exp comparative study/	
22	exp prospective study/	
23	or/21-22	
24	23 not 10	
25	23 not (11 or 19)	
26	11 or 20 or 25	
27	retina tear/	
28	(macula$ adj3 hole$).tw.	
29	or/27-28	
30	exp postoperative period/	
31	exp body position/	
32	(postur$ or position$ or prone).tw.	
33	(face adj2 down).tw.	
34	seated.tw.	

35	or/30-34	
36	29 and 35	
37	26 and 36	439

c. Search strategy in Cochrane Central Register of Controlled Trials (CENTRAL; search date: 2021/09/05)

#	Search syntax	Citations found
1	macula* near/3 hole*	
2	MeSH descriptor Retinal Perforations	
3	(#1 OR #2)	
4	MeSH descriptor Postoperative Care	
5	MeSH descriptor Postoperative Period	
6	prone or position* or postur*	
7	MeSH descriptor Prone Position	
8	(#4 OR #5 OR #6 OR #7 OR #7)	
9	(#3 AND #8)	99

Figure S2. Forrest plot of visual acuity improved rate

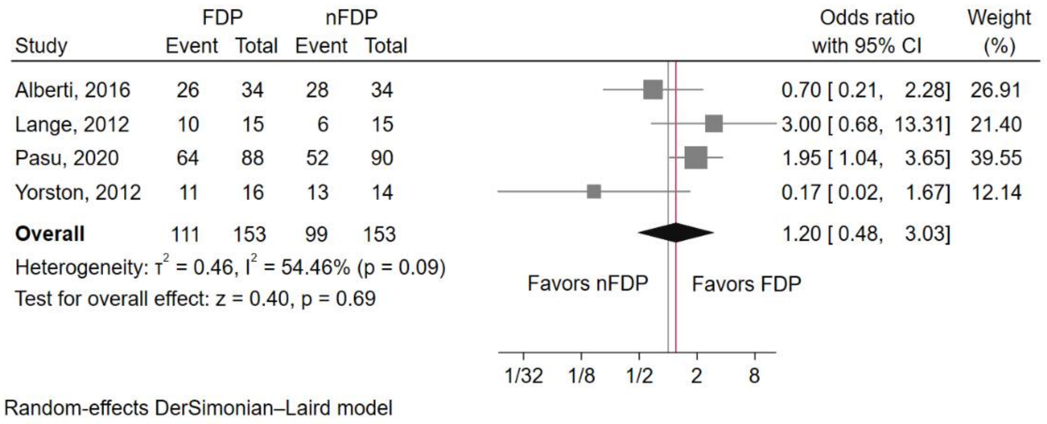

Table S3. References of excluded studies after full-text screening

Non-randomized studies:	1. Tranos PG, Peter NM, Nath R, Singh M, Dimitrakos S, Charteris D, Kon C. Macular hole surgery without prone positioning. Eye (Lond). 2007 Jun;21(6):802-6. doi: 10.1038/sj.eye.6702339. Epub 2006 Mar 31. PMID: 16575410.<br>2. Tornambe PE, Poliner LS, Grote K. Macular hole surgery without face-down positioning. A pilot study. Retina. 1997;17(3):179-85. doi: 10.1097/00006982-199705000-00001. PMID: 9196926.<br>3. Simcock PR, Scalia S. Phacovitrectomy without prone posture for full thickness macular holes. Br J Ophthalmol. 2001 Nov;85(11):1316-9. doi: 10.1136/bjo.85.11.1316. PMID: 11673297; PMCID: PMC1723751.<br>4. Malik A, Dooley I, Mahmood U. Single night postoperative prone posturing in idiopathic macular hole surgery. Eur J Ophthalmol. 2012 May-Jun;22(3):456-60. doi: 10.5301/ejo.5000039. PMID: 21928260.<br>5. Dhawahir-Scala FE, Maino A, Saha K, Mokashi AA, McLauchlan R, Charles S. To posture or not to posture after macular hole surgery. Retina. 2008 Jan;28(1):60-5. doi: 10.1097/IAE.0b013e31813c68a2. PMID: 18185139.<br>6. Bastos ALCM, Ávila MP, Isaac DLC, Aguiar LP, Aguiar AG. Surgical results regarding the correction of macular hole with and without face-down posturing using 25% SF6 gas: a retrospective case series. Arq Bras Oftalmol. 2020 Sep-Oct;83(5):361-365. doi: 10.5935/0004-2749.20200044. PMID: 33084811.<br>7. Lindtjørn B, Krohn J, Austeng D, Fossen K, Varhaug P, Basit S, Helgesen OH, Eide GE, Forsaa VA. Nonsupine Positioning after Macular Hole Surgery: A Prospective Multicenter Study. Ophthalmol Retina. 2019 May;3(5):388-392. doi: 10.1016/j.oret.2018.12.006. Epub 2018 Dec 31. PMID: 31044728.<br>8. Gotzaridis S, Liazos E, Petrou P, Georgalas I. Short-Acting Gas Tamponade with Strict Face-Down Posturing for the Treatment of Idiopathic Macular Hole. Semin Ophthalmol.

	2017;32(5):597-601. doi: 10.3109/08820538.2015.1132333. Epub 2016 Jul 1. PMID: 27367053.<br>9. Forsaa VA, Raeder S, Hashemi LT, Krohn J. Short-term postoperative non-supine positioning versus strict face-down positioning in macular hole surgery. Acta Ophthalmol. 2013 Sep;91(6):547-51. doi: 10.1111/j.1755-3768.2012.02464.x. Epub 2012 Jul 25. PMID: 22830524.<br>10. Essex RW, Kingston ZS, Moreno-Betancur M, Shadbolt B, Hunyor AP, Campbell WG, Connell PP, McAllister IL; Australian and New Zealand Society of Retinal Specialists Macular Hole Study Group. The Effect of Postoperative Face-Down Positioning and of Long- versus Short-Acting Gas in Macular Hole Surgery: Results of a Registry-Based Study. Ophthalmology. 2016 May;123(5):1129-36. doi: 10.1016/j.ophtha.2015.12.039. Epub 2016 Feb 23. Erratum in: Ophthalmology. 2017 Jun;124(6):922-923. PMID: 26920098.
Not compared face-down posture and non-face-down posture	1. Isomae T, Sato Y, Shimada H. Shortening the duration of prone positioning after macular hole surgery- comparison between 1-week and 1-day prone positioning. Jpn J Ophthalmol. 2002 Jan-Feb;46(1):84-8. doi: 10.1016/s0021-5155(01)00468-3. PMID: 11853720.<br>2. Forsaa VA, Krohn J. POSTOPERATIVE POSITIONING IN MACULAR HOLE SURGERY: An Objective Evaluation of Nonsupine Positioning and the Effect of the "Tennis Ball Technique". Retina. 2016 Jun;36(6):1081-6. doi: 10.1097/IAE.0000000000000858. PMID: 26562568.<br>3. Forsaa VA, Krohn J. AIR TAMPONADE COMBINED WITH NONSUPINE POSITIONING IN MACULAR HOLE SURGERY FOR PSEUDOPHAKIC EYES. Retina. 2017 Sep;37(9):1750-1756. doi: 10.1097/IAE.0000000000001413. PMID: 27902639.<br>4. Eckardt C, Eckert T, Eckardt U, Porkert U, Gesser C. Macular hole surgery with air tamponade and optical coherence tomography-based duration of face-down positioning. Retina. 2008 Oct;28(8):1087-96.

Same clinical trial with different reports	1. Alberti M, la Cour M. GAS-FOVEAL CONTACT: A New Approach to Evaluating Positioning Regimens in Macular Hole Surgery. Retina. 2018 May;38(5):913-921. doi: 10.1097/IAE.0000000000001654. PMID: 28463904.
Meeting abstract	1. Mark Alberti, Morten D De La Cour; Gas-foveal contact in macular hole surgery - a comparison of face-down and non-supine positioning. *Invest. Ophthalmol. Vis. Sci.* 2016;57(12):1065.
Only enrolled small idiopathic macular hole cases	1. Tadayoni R, Vicaut E, Devin F, Creuzot-Garcher C, Berrod JP, Le Mer Y, Korobelnik JF, Aout M, Massin P, Gaudric A. A randomized controlled trial of alleviated positioning after small macular hole surgery. Ophthalmology. 2011 Jan;118(1):150-5.

Figure S3. Leave-one-out sensitivity analysis of visual acuity improved rate

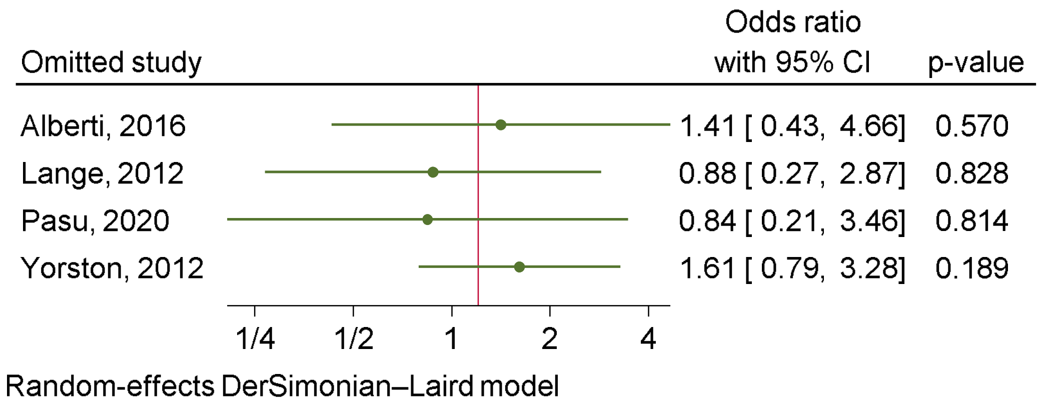

Table S3. Summary of ROB 2.0 assessment in RCTs

Study							
First author	Year	Bias arising from randomization process	Bias due to deviations from intended intervention	Bias due to missing outcome data	Bias in measurement of the outcome	Bias in selection of the reported result	Overall bias
**Alberti**	**2016**	Low	Low	Low	Low	Low	Low
**Guillaubey**	**2008**	Some concern	Low	Low	Low	Low	Some concern
**Lange**	**2012**	Low	Some concern	Low	Low	Low	Some concern
**Pasu**	**2020**	Low	Low	Low	Low	Low	Low
**Veith**	**2020**	Some concern	Low	Low	Low	Low	Some concern
**Yorston**	**2012**	Low	Some concern	Low	Low	Low	Some concern
**Zhang**	**2018**	Low	Low	Low	Low	Low	Low

Figure S4. DOI plot of large IMH closure rate

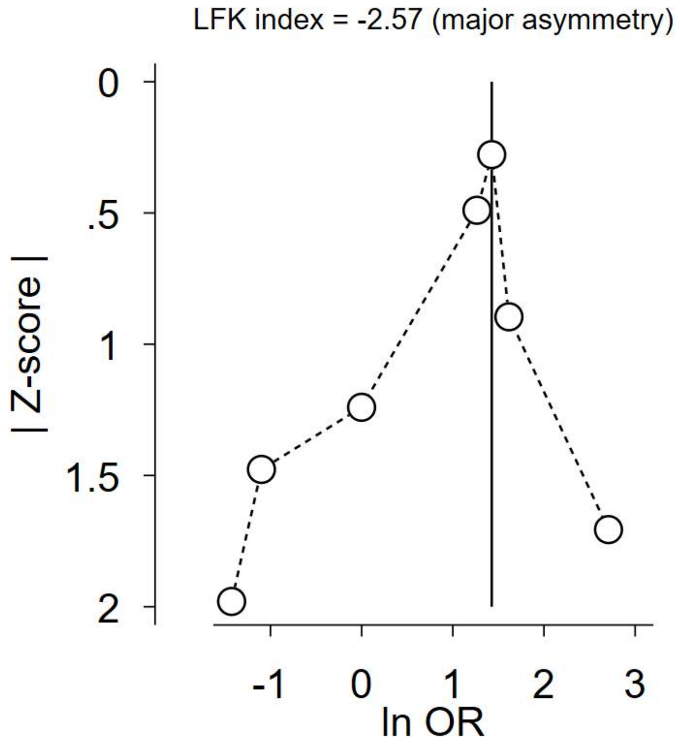

Table S5. Certainty of evidence based on GRADE (FDP vs. nFDP in large IMH surgery).

Certainty assessment							Summary of findings	
Participants (studies)	Risk of bias	Inconsistency	Indirectness	Imprecision	Publication bias	Overall certainty of evidence	Relative effect OR (95% CI)	NNTs
**IMH > 400um closure rate**								
371 (7 RCTs)	Not serious	Not serious	Not serious	Not serious	Publication bias strongly suspected[a]	⊕⊕⊕◯ **MODERATE**	3.34 (1.57 to 7.14)	7.9

GRADE, Grading of Recommendations Assessment; FDP, face-down posture; nFDP,non-face-down posture; IMH, idiopathic macular hole; NNT, number needed to treat; a, Doi plot yielded major asymmetry with LFK index of -2.57
